# Throat Swab Culture Positivity and Antibiotic Resistance Profiles in Children 2–5 Years of Age Suspected of Bacterial Tonsillitis at Hargeisa Group of Hospitals, Somaliland: A Cross-Sectional Study

**DOI:** 10.1155/2023/6474952

**Published:** 2023-04-05

**Authors:** Hamda Hussein Darod, Addisu Melese, Mulugeta Kibret, Wondemagegn Mulu

**Affiliations:** ^1^Department of Medical Laboratory Science, College of Medicine and Health Sciences, Bahir Dar University, Bahir Dar, Ethiopia; ^2^Department of Biology, Science College, Bahir Dar University, Bahir Dar, Ethiopia; ^3^Department of Biochemistry and Microbiology, Laboratory of Microbiology, Faculty of Science, Ghent University, Ghent, Belgium

## Abstract

**Introduction:**

Tonsillitis is the third most frequently diagnosed infection in the pediatric age and is associated with significant morbidity and loss of school attendance. Throat swab cultures are useful for the confirmation of children with a clinically suspected tonsillitis. However, Somaliland is one of the underdeveloped countries with a low standard of sanitation and poor health seeking culture. Treatment of tonsillitis with antibiotics is irrational and not empirical. This study determined the bacterial throat swab culture positivity and antibiotic resistance profiles of the bacterial isolates among children 2–5 years of age with suspicion of tonsillitis at Hargeisa Group of Hospital, Somaliland.

**Materials and Methods:**

A cross-sectional study was conducted from March to July 2020. A total of 374 children from 2 to 5 years of age suspicion of tonsillitis was included using a convenient sampling method. Throat swabs were collected, and bacterial isolation and identification were done using standard bacteriological procedures. Antimicrobial susceptibility testing was done using the disk diffusion method. Data on demographic variables and clinical profiles were collected using structured questionnaires. Logistic regression analysis was computed to identify factors associated with bacterial tonsillitis.

**Results:**

Overall, 120 (32.1%) (95% CI 27.4–36.8%) of children were positive for bacterial throat cultures. Of these, 23 (19.2%) were mixed bacterial isolates. The most frequent bacterial isolates were beta-hemolytic streptococci 78 (55%), *Staphylococcus aureus* 42 (29%), and *Streptococcus pneumoniae* 10 (7%). Isolates revealed 83.3–100% rate of resistance to ampicillin. Beta-hemolytic streptococci isolates were 94.9% resistant to ampicillin. *S*. *aureus* was resistant to clarithromycin (38%) while *S*. *pneumoniae* isolates were 100% resistant to ampicillin. History of tonsillitis (AOR = 0.12; 95% CI = 0.06–0.21), difficulty in swallowing (AOR = 6.99; 95% CI = 3.56–13.73), and attending schools (AOR = 2.98; 95% CI = 1.64–5.42) were found to be associated with positive throat culture.

**Conclusions:**

Resistance to ampicillin and MDR among beta-hemolytic streptococci and other isolates of throat colonizers in children with clinically suspected of bacterial tonsillitis are major concerns in Hargeisa, Somaliland. Therefore, treatments of cases are recommended to be guided by regular culture and antimicrobial susceptibility testing to prevent complications of tonsillitis and associated antibiotic resistance.

## 1. Introduction

Tonsillitis, the inflammation of the tonsils, is a contagious disease that can spread through close contact with infected persons, sharing food, drinks, and utensils. Tonsillitis impacts the health of children, the quality of life, causes significant morbidity, and loss of time for schooling [1]. Poor living conditions, exposure to environmental pollutants, and indoor air pollution are frequently reported factors associated with tonsillitis among children 2–5 years of age [2].

Although viruses are the leading cause of tonsillitis in children under the age of five, beta-hemolytic streptococci are frequently associated with symptomatic childhood bacterial tonsillitis. *Staphylococcus aureus* (*S*. *aureus*), *Streptococcus pneumoniae* (*S*. *pneumoniae*), *Haemophilus influenzae* (*H*. *influenzae*), certainly *Moraxella catarrhalis* (*M*. *catarrhalis*), *Pseudomonas aeruginosa* (*P*. *aeruginosa*), and *Klebsiella pneumoniae* (*K*. *pneumoniae*) are popular colonizers of the throat [3].

Throat swabs are neither specific nor sensitive to microbacteria causing sore throat symptoms; however, current guidelines suggest they are still useful in some cases and the clinical diagnosis of symptomatic patients' needs confirmation by throat culture and the microbiological evidence of bacteria in the throat swab proves the existence of bacteria in the swab site [4].

The emergence of drug-resistant bacteria in tonsillitis is getting higher every year. Throat colonization with methicillin resistant *S*. *aureus* (MRSA) is frequent in children. Irrational use of antibiotics by humans, production of beta-lactamase enzymes, and the formation of biofilms by pathogens are the main reasons for the emergence of drug resistance [5]. The spread of drug-resistant bacteria has led to treatment failure and the recurrence of tonsillitis among children with poor sanitation and hygiene in underdeveloped countries. The situation is critical in Somaliland, where antimicrobials are vastly and frequently used irrationally [6]. This might increase the emergence of resistance to commonly used antibiotics for the treatment of tonsillitis.

Like other similar health settings in Somaliland, routine culture and antibiotic susceptibility tests are not usually performed as essential parts of patient care in Hargeisa Group of hospitals and treatments are mostly empirical. Published data on throat culture and antimicrobial resistance profiles of bacteria, as well as the associated factors in children suspicion of tonsillitis, are limited in Somaliland. Pathogen occurrence and susceptibility profiles show substantial geographic variations, as well as significant differences in various populations and environments [7, 8]. Asymptomatic children can be the sources of dissemination of bacteria causing tonsillitis to noninfected children at home or at school settings [5]. These can lead to a wide range of tonsillar infections. Thus, knowledge of the local bacterial isolate and susceptibility profiles is required to detect on time any changes that might have occurred so that appropriate recommendations for optimal empirical therapy of bacterial infections can be made.

Therefore, we present the first report of the profiles of bacterial throat culture isolates and antibiotic resistance and associated factors in children 2–5 years of age with suspicion of bacterial tonsillitis at Hargeisa Group of Hospital, Somaliland.

## 2. Materials and Methods

### 2.1. Study Design, Period, and Setting

A hospital-basedcross-sectional study was conducted between March and July 2020 in Hargeisa Group of Hospital (HGH), Somaliland. Hargeisa Group of Hospital is located in Maroodi Jeex Region, the capital city of Somaliland known as Hargeisa. According to the 2019 census report from the Central Statistics Department of Somaliland, Hargeisa has a total population of 1.2 million. Hargeisa Group of Hospital is the largest referral public hospital with more than 200 healthcare professionals. It is one of the health hubs in Somaliland. Daily, 50 outpatients and 1 to 4 hospitalized children attend the Pediatric Clinic for different medical conditions. Children with tonsillitis are diagnosed clinically and managed without the guidance of culture and antimicrobial susceptibility testing. All children aged 2–5 years suspicion of tonsillitis at ear, nose, and throat (ENT) of HGH were the study population.

### 2.2. Inclusion and Exclusion Criteria

Children 2–5 years of age with either sore throat or swollen tonsils, difficulty swallowing, white pus-filled spots on the tonsils, or swollen lymph nodes with or without fever (temperature >38°C at presentation) were considered symptomatic for bacterial tonsillitis [4]. On the other hand, children who were on antibiotics 2 weeks prior to recruitment or had tonsillectomy were excluded from the study.

### 2.3. Sample Size and Sampling Procedure

The sample size was calculated using the single population formula *n* = (*Zα*/2)^2^*P* (1 − *P*)/*d*^2^, where *n* = sample size, *Z* = level of confidence according to the standard normal distribution, *P* = sample proportion, and *d* = tolerated margin of error. Therefore, by taking *Z* (*α*/2) = 1.96 for a level of confidence of 95%, *P*=0.5, which is the maximum proportion of positive throat cultures and 5% margin of error, the sample size was calculated as *n* = (1.96)^2^ × 0.5 (1 − 0.5)/(0.05)^2^ = 384. All children 2–5 years of age suspected of bacterial tonsillitis attending at ENT Department of HGH, and who fulfilled the inclusion criteria were included consecutively until the sample size was reached. However, due to the lack of sufficient throat swabs and incomplete questionnaires, only 374 children aged from 2 to 5 years suspected of bacterial tonsillitis participated in the study.

### 2.4. Variables

Bacterial throat culture positivity was the dependent variable while demographic, clinical, and other explanatory variables were independent variables.

### 2.5. Data Collection

A structured questionnaire was used to collect data on demographic characteristics, clinical profiles, and other variables. Data on child's age, mother's age, father's age, gender, residence, maternal and paternal education, parental occupation, type of breast feeding, attending daycare and school, living in overcrowded environments, and exposure to wood biofuels were collected with face-to-face interviews of their caregiver using a structured questionnaire. Moreover, clinical information such as history of contact with someone who had cough, history of tonsillitis, the current type of tonsillitis, number of previous tonsillitis, body temperature, sore throat, swollen tonsils, headache, swollen lymph nodes, difficulty in swallowing, white exudates on the throat, weight loss, tonsillar structural change, and history of drug use) were collected by the attending pediatricians.

### 2.6. Throat Swab Sample Collection and Processing

Throat swabs were taken by the attending pediatricians from each patient using a sterile cotton swab. Visible exudates or hyperemic areas on the tonsillar walls were swabbed with a sterile cotton swab, while the tongue was depressed by a wooden spatula when necessary. All swab samples were immediately transported to the Microbiology Department of HGH using Amie's transport medium (Oxoid, England). Swabs were simultaneously plated onto Tryptic Soy Agar (Himedia, India) containing 5% sheep blood, chocolate agar (CA), and MacConkey (MAC) Agar (Himedia, India) and incubated for 48 h at 37°C. Chocolate agar was incubated in a candle jar to get 5% CO_2_, while BA and MAC were incubated under a normal atmosphere.

### 2.7. Identification of Bacterial Isolates

Pure colonies of the bacterial isolates were identified to the species level following standard enzymatic and biochemical tests [9]. White to grey large or small colony forming units with a zone of beta hemolysis around 2-3 mm in diameter surrounding each colony plus, Gram positive cocci arranged in a chain and were both coagulase and catalase negative were taken as beta-hemolytic streptococci isolates. Small, shiny, and translucent colonies surrounded by a zone of alpha hemolysis on BA and were Gram positive and susceptible to optochin were identified as *S. pneumoniae* isolates. *S. aureus* isolates were identified by Gram positive clusters forming glistering golden yellow colonies on BA and mannitol salt agar (MSA) which were coagulase, catalase, and oxidase positive. *Moraxella catarrhalis* were identified by nonhemolytic grey to white colonies on blood agar (BA) which were oxidase, and catalase positive. *K*. *pneumoniae* and *P. aeruginosa* isolates were identified by standard biochemical tests.

### 2.8. Antimicrobial Susceptibility Testing

Susceptibilities of all identified bacterial isolates to different antimicrobials were performed on Mueller-Hinton agar (MHA) containing 5% sheep's blood (Himedia, India) according to the criteria of the 2019 Clinical and Laboratory Standards Institute (CLSI) (10) using the Kirby-Bauer disk diffusion method. The following drug discs were tested: ampicillin (10 *μ*g), amoxicillin-clavulanic acid (20/10 *μ*g), cefoxitin (30 *μ*g), gentamicin (10 *μ*g), clarithromycin (15 *μ*g), erythromycin (15 *μ*g), ofloxacin (5 *μ*g), and ciprofloxacin (5 *μ*g). These antibiotic discs were selected based on the frequent prescriptions of these drugs for the treatment of tonsillitis infection in the study area and using the CLSI guideline recommendations [10]. A loop full of culture was taken from a pure culture colony and transferred to a tube containing 5 ml of normal saline and mixed gently until it forms a homogenous suspension. The turbidity of the suspension was then adjusted to the turbidity of McFarland 0.5 (which carries 10^8^ CFU/ml) and was swabbed on a dry surface of MHA plate with 5% sheep blood (150 mm) using a sterile cotton swab. Antibiotic discs were dispensed using a single disc dispenser. Plates were then incubated for 24 h at 37°C. Diameters of the zone of inhibition around the discs were measured using a digital caliper. The results of the zone of inhibition of antibiotics were interpreted based on the 2019 CLSI guideline [11]. All *S*. *aureus* isolates were subjected to cefoxitin disc diffusion test on Mueller-Hinton agar plates. Plates were incubated at 35°C for 18 h and inhibition zones with a diameter of ≤21 mm were reported as methicillin resistant and ≥22 mm considered as methicillin sensitive. Bacterial isolates that revealed acquired nonsusceptibility to at least one agent in three or more antibiotic categories were considered MDR [10].

### 2.9. Quality Control

Data collectors were trained on the aim of the study and data collection procedures. The completeness of data was also checked. The proper functioning of materials, equipment, culture media, and procedures were checked. Specimens were collected following standard bacteriological procedures. To prevent contamination, all throat swabs were analyzed within two hours of collection. Culture media were checked for sterility by incubating 5% of each batch of the medium at 37°C for 24 hrs. The performance of all prepared culture media was checked by inoculating with the American Type Culture Collection (ATCC) standard reference strains *S*. *aureus* (ATCC 29213), *S*. *penumoniae* (ATCC 49618), and *P*. *aeruginosa* (ATCC 27853).

### 2.10. Data Analysis

Data were entered and analyzed using SPSS version 25 (IBM Corp, Armonk, NY, USA). Univariate analysis was made to generate summary values for the most important variables. Logistic regression analysis was made to determine the association between dependent and independent variables. The generated data were compiled with frequency tables and other statistical summary measures. A stepwise logistic regression model was used to find factors associated with culture positive bacterial tonsillitis and statistical significance was set at *P* < 0.05.

## 3. Results

### 3.1. Characteristics of the Study Participants

A total of 374 children aged 2–5 years suspicion of bacterial tonsillitis participated in the study, making a response rate of 97.4% (374/384). Among them, 200 (53.5%) were males. Most (305, 81.6%) of the children were urban residents. The age range of the children was 2 to 5 years. The majority (141, 37.7%) of the children were five years old. The age of children's mother ranged from 20 to 45 years. Most (69%) of the parents were employees (Table 1).

### 3.2. Isolation Rate of Bacterial Throat Culture

Overall, 120 (32.1%) of the children were positive for bacterial throat culture. The proportion of throat culture positivity was higher in males (73, 36.5%) than in females (47, 27%). It was higher in urban (101, 33.1%) than rural (19, 27.5%) residents. The percentage of throat culture positivity was higher (78, 41.9%) in children from mothers who are unable to read and write than in the other groups (5.9–26.7%). Children from fathers who had higher educational attainment had the lowest percentage of throat culture positivity compared to others (Table 1).

### 3.3. Bacterial Throat Culture and Clinical Profiles

Table 2 depicts the results of bacterial throat culture with clinical profiles among children 2–5 years of age. Most children had presented with acute tonsillitis 202 (54%) and sore throat (343, 91.7%). Swollen tonsils were presented in 367 (98.1%) of children. On the other hand, swollen lymph nodes were presented in 151 (40.4%) children. Moreover, 147 (39.3%) and 69 (18.4%) of the children had difficulty swallowing and white exudates, respectively (Table 2).

Of the total, 172 (46%) of the children were positive for bacterial throat culture. The percentage of positive bacterial throat cultures was higher among children with a history of tonsillitis 95 (55.2%) than the others 25 (12.4%). The percentage of throat culture positivity was the highest (30, 57.7%) in children with symptoms of chronic tonsillitis. Children with swollen tonsils had a higher percentage of throat culture positivity rates (119, 32.4%) than those without swollen tonsils (1, 14.3%). The proportion of throat culture isolation was higher among children who had weight loss (67, 38.2%) than their counterparts (53, 61.8%) (Table 2).

### 3.4. Bacterial Throat Culture in Relation to Other Variables

Overall, 96 (25.7%) and 228 (61%) of the children were exclusively breastfed and had a history of contact with coughing patients, respectively. On the other hand, 86 (23%) and 282 (75.4%) of the children were daycare center attendees and school attendees, respectively. Most of the children lived in a crowded houses (268, 71.7%) and 331 (88.5%) had exposure to biofuels (Table 3).

The percentage of positive bacterial throat cultures was higher among children who had a history of contact with coughing patients (85, 37.3%) than their counterparts (35, 24%). Daycare center attendee children had a higher (34/86, 39.5%) percentage of bacterial isolation than others 86 (29.9%). Moreover, school-attending children had a higher (48, 52.2%) percentage of bacterial isolation than their counterparts (72, 25.5%). The proportion of bacterial isolation was higher among children who had exposure to biofuels (117, 35.3%) than their counterparts (3, 7%) (Table 3).

### 3.5. Distribution of Bacteria Isolates

**Figure 1:**
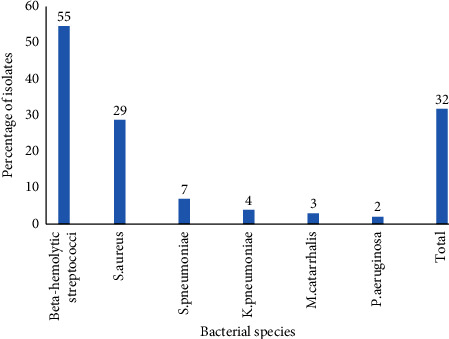
Frequency of bacterial species isolated from children 2 to 5 years of age with suspicion of bacterial tonsillitis at Hargeisa Group of Hospital.

A total of 143 (32%) bacterial pathogens were isolated from 120 culture positive samples. The most frequent isolate was beta-hemolytic streptococci 78 (55%) followed by *S. aureus* 42 (29%) and *S. pneumoniae* 10 (7%) (Figure 1).

Among the 120 children positive for bacterial throat culture, 23 (19.2%) had mixed bacterial isolates. Beta-hemolytic streptococci and *S*. *aureus*, *S*. *pneumoniae* and *S*. *aureus,* and *S*. *aureus* and *M*. *catarrhalis* were the most common mixed isolates with a proportion of 10 (8.3%), 4 (3.3%), and 4 (3.3%), respectively (Table 4).

### 3.6. Antibiotic Resistance Profiles of Bacterial Isolates

Overall, 137 (91.9%) bacterial isolates were resistant to ampicillin. Relatively higher resistance percentages were found to gentamicin (41.3%), ofloxacin (34.3%), and clarithromycin (32.2%). Beta-hemolytic streptococci*, S. aureus*, *S. pneumoniae*, and *M. catarrhalis* isolates revealed an overall resistance of 33.5%, 37.2%, 28.8%, and 50%, respectively. Beta-hemolytic streptococci revealed resistance to ampicillin (94.9%), ofloxacin (43.6%), and gentamicin (42.3%). *S. aureus* isolates showed resistance to ampicillin (83.8%), clarithromycin (38.1%), and ciprofloxacin (35.7%). The proportion of MRSA was 19 (45.2%). The percentage of *S. pneumoniae* isolates resistant to ampicillin, gentamicin, clarithromycin, and erythromycin was 100%, 60%, 30%, and 30%, respectively. All *P*. *aeruginosa* isolates were resistant to ciprofloxacin and ampicillin (Table 5).

### 3.7. Multiple Drug Resistant (MDR) Profiles of Bacterial Isolates

Multidrug resistance (MDR) is the resistance of a bacterial isolate to three or more antibiotics taken from different categories. Overall, 71 (49.7%) of the bacterial species were MDR and 52.6% of beta-hemolytic streptococci were MDR. The MDR profile of *S*. *aureus and S. pneumoniae* isolates were 17 (40.5%) and 6 (60%), respectively (Table 6).

### 3.8. Multivariable Analysis

Based on multivariable analysis, positivity for bacterial throat culture was significantly associated with difficulty in swallowing (AOR = 6.99, CI = 3.56–13.13), weight loss (AOR = 0.33, CI = 0.186–0.597), attending school (AOR = 2.98, CI = 1.64–5.42), history of tonsillitis (AOR = 0.12, CI = 0.06–0.21), and exposure to biofuel (AOR = 0.19, CI = 0.04–0.84). Children who had difficulty swallowing were 7 times more likely to become positive for bacterial throat culture, compared to children who did not have difficulty swallowing. Likewise, school-attending children were 3 times more likely to be positive for bacterial throat culture compared to nonattenders. Children with a history of tonsillitis were more likely to have a positive bacterial throat culture than those without a history of tonsillitis. Similarly, children who had weight loss and exposure to biofuels were more likely to become positive for throat culture compared to those who did not have weight loss and exposure to biofuels (Table 7).

## 4. Discussion

Tonsillitis has a considerably negative impact on the patients' quality of life and has a significant burden on public health. Untreated childhood tonsillitis leads to peritonsillar abscess, tonsillar stones, and rheumatic fever. Therefore, identification of bacterial isolates and determination of antibiotic susceptibility profiles from throat a swab of children with suspicion of bacterial tonsillitis is useful for the treatment of tonsillitis in the healthcare setting of Somaliland where patients are treated without a routine culture diagnosis. Therefore, this study presents the first report of the antibiotic resistance profiles of bacterial isolates from throat swab cultures of children with suspicion of bacterial tonsillitis in HGH.

In this study, 32.1% of children 2–5 years of age were positive for bacterial throat culture. Due to lack of previously published data in Somaliland, a comparison of countrywide results was not possible. However, the prevailing magnitude of bacterial throat swab culture is higher than similar studies with a prevalence of 11.3% in Ethiopia [12], 20.6% in Tanzania [13], 21.6% in Norway [14], and 19% in Bangladesh [15]. The prevalence of positive bacterial throat swab culture in this study was lower than studies done in the United Kingdom (79%) [16], Trinidad (62.5%) [17], India (72%) [18], Saudi Arabia (65%) [19], Benin (73.97%) [20], and Ethiopia (51%) [21]. The lower rate of positive bacterial throat culture in the present study compared to other developing countries might be attributed to differences in geography, community living status and hygienic practices, host factor, and educational level of the parents.

The proportion of positive bacterial throat swab culture in children 2–5 years of age was higher in males than in females, which is similar to studies from India [2] and Nigeria [22]. Moreover, the percentage of positive bacterial throat cultures was higher among children living in urban than rural areas in the present study. This was similar to studies done in India [2] and Ethiopia [21]. This might be due to variation in encountering infected or colonized people, exposure to air pollution from biofuel use, schooling, and house crowding.

A high rate of positive for bacterial throat swab culture was reported among patients symptomatic of chronic (57.7%) and recurrent tonsillar infections (51.2%). These are indications of antimicrobial resistance and tonsillectomy. Although we did not differentiate the pyogenic group of streptococci from the anginosus group due to limitations in the laboratory facility, beta-hemolytic streptococci were the most frequent isolate from children with suspicion of bacterial tonsillitis in this study and its percentage (55%) is similar with studies in Ohio (58%) [23], Italy (69%) [24], and Trinidad (82.2%) [17]. However, it is higher than studies from Egypt (17%) [25], Iran (20%) [26], Iraq (29.7%) [27], India (22.25%) [28], Saudi Arabia (40%) [29], and Ethiopia (12.2%) [30]. This variation might be influenced by the climate, age, and geographical location of the study participants.

In the present study, *Staphylococcus aureus* was the second most frequent isolate of throat swab cultures from children with suspicion of bacterial tonsillitis with a rate of 29%. This could be due to the persistence of *S. aureus* in the tonsillar tissues, treatment with antimicrobials, and antibiotic resistance. Moreover, *S*. *aureus* has the potential to form biofilm which results in recurrent and chronic infection as well as treatment failure. The isolation of *S. aureus* as the main agent of tonsillitis has been reported by several authors in Ethiopia [19], Brazil (40%) [31], Trinidad (68.9%) [17], and Nigeria (32.1%) [22].

It is a fact that isolation of *S*. *pneumoniae* indicates the existence of recurrent tonsillitis in children. The percentage (7%) of *Streptococcus pneumoniae* isolates from children with suspicion of bacterial tonsillitis in the present study is lower than studies done from Poland (14%) [32], Belgium (21%) [33], Italy (4%) [34], and South Ethiopia (62.5%) [35]. However, it was higher than studies done in the US (3.5%) [36], Nepal (4%) [37], and Nigeria (3.3%) [38]. The percentage (4%) of *Klebsiella pneumoniae* isolates in this study is higher than a study done in Brazil (1.4%) [39], but was lower than studies done in Singapore (6.6%) [40] and Indonesia (7%) [41]. In this study, the prevalence of *Moraxella catarrhalis* was 3% which is different from studies done in the USA (22%) [42], Brazil (28.5%) [43], Denmark (53%) [44], Tanzania (90.8%) [45], and Ethiopia (12.3%) [21].

In the present study, there is a high proportion of mixed isolates particularly with beta-hemolytic streptococci and *S. aureus*, *S*. *pneumoniae* and *S*. *aureus*, and *S*. *pneumoniae* and *M*. *catarrhalis*. This co-colonization of the tonsils may contribute to the severe inflammatory process and the failure of penicillin and ampicillin therapy, which finally results in recurrent infection, tonsillectomy, rheumatic fever and other complications [37, 41].

In this study, the percentage of MRSA isolates among children with suspicion of bacterial tonsillitis was 45.2%. This is higher than studies done in Germany (0.8%) [46], Lahore (5.5%) [47], Japan (0.8%) [48], Brazil (3.3%) [31], Ethiopia (2.3%) [49], USA (16%) [50], Benin (17.95%) [20], and Uganda (32%) [51]. The highest proportion of MRSA in the present study compared to others might be over prescription and unnecessary use of antibiotics for various clinical conditions and outpatient care. Moreover, the uncontrolled contact of children with hospitalized patients who could have contracted the MRSA from the hospital might be the possible reason.

The resistance of the isolates to ampicillin was 91.6% and 14.7% for the association of amoxicillin and clavulanate. The higher resistance to ampicillin by all bacterial isolates might be due to the production of beta-lactamase enzyme, as well as abuse and excessive use of cheap drugs, which can be afforded and administered without culture diagnostic guidance. This is a major concern that limits the use of this common therapeutic option in clinical practice in developing countries. The rate of ampicillin resistance is comparable to reports from Nigeria (100%) [20, 52].

Although the existence of anginosus streptococci group is more likely, the resistance rate of 94.9% of beta-hemolytic streptococci to ampicillin is worrisome as B-lactam antibiotics are the drug of choice for strep throat. Therefore, further study on the molecular characterization of species of *Streptococcus* from children with throat swab culture is recommended. The percentage of beta-hemolytic streptococci resistant to gentamicin (42.3%) and ofloxacin (43.6%) in the present study was comparable to studies done in Iran (32.2%) [53]. This likely is due to the enzymatic inactivation mediated by aminoglycoside-modifying enzymes (AMEs) and point mutations in the quinolone resistance-determining region (QRDR).

The resistance of *S. aureus* to ciprofloxacin (35.7%) in this study was lower than studies done in Egypt (90.9%) [28] and Nepal (100%) [54], but was higher than studies done in Brazil (24.6%) [31]. On the other hand, the resistance profile of *S. pneumoniae* to erythromycin (30%) is similar to a study done in Malaysia, with a rate of (30%) but different from studies done in China (56%, 20%) [55], Lithuania (78.8%) [56], and Ethiopia (12.4%) [57].

One of the major worries when determining the resistance profiles of isolates is the availability of MDR strains. In this study, half of the bacterial isolates were MDR. This is a serious problem for children 2–5 years of age in Somaliland. Children involved in the study area were outpatients and they might have constant contact with other children and their families. Moreover, in the study area, there is no routine culture and antimicrobial susceptibility testing and management of children with tonsillitis are empirical. These may result from repeated infections of the tonsils, pyogenic meningitis, rheumatic fever, lower respiratory tract infections, and difficulty to select effective antibiotics. Furthermore, the existence of MDR isolates demonstrates the persistence of the bacteria and the possibility of antimicrobial resistance, dissemination, and recurrence of infection [37].

The percentage of MDR *S. pneumoniae* (60%) in this study was higher than studies from Poland (52.9%) [58], Lithuania (12.5%) [56], and Vietnam (35%) [59]. In this study, all isolates of *Pseudomonas aeruginosa* were MDR (100%), which is concurrent with a study in Brazil (100%) [39]. These high proportions of MDR among the isolates might be due to productions of beta-lactamase enzyme by *Pseudomonas aeruginosa* and the production of penicillin binding proteins in *Streptococcus pneumoniae*.

In the present study, difficulty in swallowing is one of the predictors for positive bacterial throat culture in children with suspicion of bacterial tonsillitis. Similar findings were reported in India [2] and Lithuania (48). History of tonsillitis was also a predictor variable in this study, which was similar to studies done in Ethiopia [53], and Yemen [55]. These might be due to cohabitations of the tonsils by multiple bacterial isolates as depicted in Table 4 and failure of penicillin and ampicillin therapies.

Weight loss was also another predictor for bacterial tonsillitis in this study in which similar studies were reported in Iran [54] and Germany [58]. Furthermore, attending school was a risk factor for tonsillitis in this study similar to studies done in Uganda [57]. This might be due to overcrowding during schooling among children where carrier children can easily interact with healthy children.

### 4.1. Limitations of the Study

This study provided the first report of data on the profile of bacterial throat cultures and their resistance to antibiotics from children 2 to 5 years of age with suspicion of bacterial tonsillitis at Hargeisa Group of Hospital. However, the study was limited to identifying nonbacterial causes of tonsillitis. Due to the limited resources, colonizations and causes were not differentiated and bacterial isolates were not identified with molecular techniques like PCR and MALDI-TOF MS. Furthermore, bacterial isolates resistant to ampicillin were not further confirmed by minimum inhibitory concentrations (MIC). Therefore, the interpretive conclusions and recommendations of this study should be based on the findings of isolation and phenotypic identification methods.

## 5. Conclusions

A high prevalence of positive bacterial throat swab cultures resistant to different antibiotics, MRSA, and mixed isolates was found. Beta-hemolytic streptococci followed by *S. aureus* and *S. pneumoniae* were the most frequent isolates. Most of the bacterial isolates were resistant to ampicillin. However, amoxicillin-clavulanic acid and ciprofloxacin are the least resistant drugs. Therefore, the result points out that treatment tonsillitis due to bacteria guided by throat swab culture and antimicrobial susceptibility testing. Further investigations to differentiate colonization and pathogens, identify nonbacterial causes of tonsillitis, differentiate species of beta-hemolytic, conduct studies covering larger geographical areas to draw the magnitude, and topographic variations are needed to control the spread of tonsillitis among children within five years of age.

## Figures and Tables

**Table 1:** Bacterial throat culture positivity among children 2–5 years of age with suspicion of bacterial tonsillitis at Hargeisa Group of Hospital (*N* = 374).

Variables	Total	Culture results		COR (95% CI)	*P* value
	*N* (%)	Positive	Negative		
		*N* (%)	*N* (%)		
*Children age (years)*					
2	2 (0.5)	2 (100)	0	NA	0.199
3	110 (29.4)	36 (32.7)	74 (67.3)		
4	121 (32.4)	40 (33.1)	81 (66.9)		
5	141 (37.7)	42 (29.8)	99 (70.2)		

*Gender*					
Male	200 (53.5)	73 (36.5)	127 (63.5)	0.64 (0.41–1.00)	0.05
Female	174 (46.5)	47 (27)	127 (73)		

*Residence*					
Rural	69 (18.4)	19 (27.5)	50 (72.5)		0.37
Urban	305 (81.6)	101 (33.1)	204 (66.9)	0.78 (0.43–1.37)	

*Father's education*					
Not able to read and write	55 (14.7)	18 (32.7)	37 (67.3)	3.46 (0.67 = 17.72)	0.002
Able to read and write	56 (15)	29 (51.8)	27 (48.2)	2.53 (0.46–13.79)	
Primary school	81 (21.7)	19 (23.5)	62 (76.5)	1.01 (0.20–5.57)	
High school	82 (21.9)	30 (36.6)	52 (63.4)	0.9 (0.16–5.04)	
Higher education	100 (26.7)	24 (24)	76 (76)		

*Mother's education*					
Not able to read and write	186 (49.7)	78 (41.9)	108 (58.1)	NA	0.001
Able to read and write	105 (28.1)	28 (26.7)	77 (73.3)		
Primary school	36 (9.6)	7 (19.4)	29 (80.6)		
High school	30 (8)	6 (20)	24 (80)		
Higher education	17 (4.5)	1 (5.9)	16 (94.1)		

*Parental occupation*					
Unemployed	116 (31)	26 (22.4)	90 (77.6)	1.98 (1.19–3.29)	0.020
Employed	258 (69)	94 (36.4)	164 (63.6)		

*Mother's age (years)*					
20–25	98 (26.2)	26 (26.5)	72 (73.5)	NA	0.14
26–30	169 (45.2)	59 (34.9)	110 (65.1)		
31–35	90 (24.1)	27 (30)	63 (70)		
36–40	15 (4)	6 (40)	9 (60)		
41–45	2 (0.5)	2 (100)	0 (0)		

Total	374 (100)	120 (32.1)	254 (67.9)		

*Key.* NA: not applicable.

**Table 2:** Bacterial throat culture positivity and clinical profiles of children with suspicion of bacterial tonsillitis at Hargeisa Group of Hospital.

Variables	Culture results			COR (95% CI)	*P* value
	Total	Positive	Negative		
	*N* (%)	*N* (%)	*N* (%)		
*History of tonsillitis*					
Yes	172 (46)	95 (55.2)	77 (44.8)	8.72 (5.22–14.63)	<0.001
No	202 (54)	25 (12.4)	177 (87.6)		

*Number of previous tonsillitis*					
None	202 (54)	26 (12.9)	176 (87.1)		
One	15 (4)	9 (60)	6 (40)	8 (4.63–13.82)	<0.001
Two	24 (6.4)	14 (58.3)	10 (41.7)	1.38 (0.44–4.35)	0.58
Three	13 (3.5)	6 (46.2)	7 (58.3)	0.84 (0.35–2.1)	0.71
≥Four	120 (32.1)	65 (54.2)	55 (45.8)	0.79 (0.26–2.35)	0.67

*Type of tonsillitis*					
Acute	202 (54)	25 (12.4)	177 (87.6)		
Chronic	52 (13.9)	30 (57.7)	22 (42.3)	0.11 (0.68–0.19)	<0.001
Recurrent	120 (32.1)	65 (54.2)	55 (45.8)		

*Weight loss*					
Yes	143 (38.2)	67 (38.2)	76 (46.9)	2.96 (1.89–4.64)	<0.001
No	231 (61.8)	53 (61.8)	178 (22.9)		
	374 (100)	120 (32.1)	254 (67.9)		

*Tonsillar structural change*					
Yes	52 (13.9)	30 (57.7)	22 (42.3)	3.52 (1.93–6.42)	<0.001
No	322 (86.1)	90 (28)	232 (72)		
	374 (100)	120 (32.1)	254 (67.9)		

*Body temperature*					
37°C	42 (11.2)	12 (28.6)	30 (71.4)	1.21 (0.59)	0.61
≥38°C	332 (88.8)	108 (32.5)	224 (67.5)	1.21 (0.59)	
	374 (100)	120 (32.1)	254 (67.9)		

*Sore throat*					
Yes	343 (91.7)	111 (32.4)	232 (63.6)	0.86 (0.38–1.92)	0.704
No	31 (8.3)	9 (29)	22 (71)		
	374 (100)	120 (32.1)	254 (67.9)		

*Swollen tonsillitis*					
Yes	367 (98.1)	119 (32.4)	248 (67.6)	0.35 (0.04–2.92)	0.308
No	7 (1.9)	1 (14.3)	6 (85.7)		

*Headache*					
Yes	105 (28.1)	33 (31.4)	72 (68.6)	1.04 (0.64–1.69)	0.865
No	269 (71.9)	87 (32.3)	182 (67.7)		

*Swollen lymph nodes*					
Yes	151 (40.4)	53 (35.1)	98 (64.9)	1.26 (0.81–1.96)	0.304
No	223 (59.6)	67 (30)	156 (70)		

*White exudates*					
Yes	69 (18.4)	19 (27.5)	50 (72.5)	0.77 (0.43–1.37)	0.370
No	305 (81.6)	101 (33.1)	204 (66.9)		

*Difficulty of swallowing*					
Yes	147 (39.3)	27 (18.4)	120 (81.6)	0.33 (0.19–0.53)	<0.001
No	227 (60.7)	93 (41)	134 (59)		
Total	374 (100)	120 (32.1)	254 (67.9)		

**Table 3:** Bacterial throat culture and other explanatory variables of children with 2–5 years of age with suspicion of bacterial tonsillitis at Hargeisa Group of Hospital (*N* = 374).

Variables	Culture result			COR (95% CI)	*P* value
	Total	Positive	Negative		
	*N* (%)	*N* (%)	*N* (%)		
*Contact with cough patients*					
Yes	228 (61)	85 (37.3)	143 (62.7)	1.89 (1.18–3.00)	0.008
No	146 (39)	35 (24)	111 (76)		

*Breast feeding*					
Mixed	278 (74.3)	91 (32.7)	187 (67.3)	1.12 (0.68–1.86)	0.65
Exclusive	96 (25.7)	29 (30.2)	67 (69.8)		

*Daycare centre attendee*					
Yes	86 (23)	34 (39.5)	52 (60.5)	1.54 (0.93–2.53)	0.092
No	288 (77)	86 (29.9)	202 (70.1)		

*Living in overcrowded house*					
Yes	268 (71.7)	76 (28.4)	192 (71.6)	0.56 (0.35–0.89)	0.014
No	106 (28.3)	44 (41.5)	62 (58.5)		

*Exposure to wood biofuel*					
Yes	331 (88.5)	117 (35.3)	214 (64.7)	7.29 (2.21–24.1)	0.001
No	43 (11.5)	3 (7)	40 (93)		
	374 (100)	120 (32.1)	254 (67.9)		

*School attendee*					
Yes	92 (24.6)	48 (52.2)	44 (47.8)	0.31 (0.19–0.51)	<0.001
No	282 (75.4)	72 (25.5)	210 (74.5)		

Total	374 (100)	120 (32.1)	254 (67.9)		

**Table 4:** Distribution of mixed isolates and MRSA from the total confirmed bacterial throat culture (*n* = 120) in children 2–5 years of age with suspicion of bacterial tonsillitis at Hargeisa Group of Hospital.

Type of mixed isolates	Frequency (%)
Beta-hemolytic streptococci *+* *S. aureus*	10 (8.3)
Beta-hemolytic streptococci *+* *P. aeruginosa*	3 (2.5)
*S. pneumoniae* *+* *S. aureus*	4 (3.3)
*S. pneumoniae* *+* *K. pneumoniae*	2 (1.7)
*S. aureus* *+* *M. catarrhalis*	4 (3.3)

Total	23 (19.2)

MRSA	19 (15.8)

*Key.* MRSA: methicillin resistant *S*. *aureus*.

**Table 5:** Antibiotic resistance profile of isolates from children 2 to 5 years of age with suspicion of bacterial tonsillitis at Hargeisa Group of Hospital.

Antibiotics tested	Beta-hemolytic streptococci		*S*. *aureus*		*S*. *pneumoniae*		*M. catarr halis*		*P*. *aeruginosa*		*K*. *pneumoniae*		Total	
	(*n* = 78)		(*n* = 42)		(*n* = 10)		(*n* = 4)		(*n* = 3)		(*n* = 6)		(*n* = 143)	
	^#^T	R%	^#^T	R%	^#^T	R%	^#^T	R%	^#^T	R%	^#^T	R%	^#^T	R%
Cefoxitin	NA	NA	42	19 (45.2)	NA	NA	NA	NA	NA	NA	NA	NA	NA	NA
Amoxicillin-clavulanic acid	78	6 (7.7)	42	10 (23.8)	10	0	4	2 (50)	3	1 (33.3)	6	2 (33.3)	143	21 (14.7)
Ciprofloxacin	78	8 (10.3)	42	15 (35.7)	10	0	4	2 (50)	3	3 (100)	6	2 (33.3)	143	30 (21)
Clarithromycin	78	22 (28.2)	42	16 (38.1)	10	3 (30)	4	2 (50)	3	1 (33.3)	6	2 (33.3)	143	46 (32.2)
Gentamicin	78	33 (42.3)	42	13 (31)	10	6 (60)	4	3 (75)	3	2 (66.7)	6	2 (33.3)	143	59 (41.3)
Ofloxacin	78	34 (43.6)	42	11 (26.2)	10	1 (10)	4	0	3	2 (66.7)	6	1 (16.7)	143	49 (34.3)
Erythromycin	78	22 (28.2)	42	11 (26.2)	10	3 (30)	4	3 (75)	3	2 (66.7)	NA	NA	137	41 (29.9)
Ampicillin	78	74 (94.9)	42	35 (83.3)	10	10 (100)	4	4 (100)	3	3 (100)	NA	NA	137	126 (91.9)
Total	546	199 (36.4)	294	111 (37.8)	70	23 (32.9)	28	16 (57.1)	21	14 (66.7)	30	9 (30)	989	372 (37.6)

^#^T: number of isolates tested and R%: percent of isolates resistant to antimicrobial agents.

**Table 6:** Multidrug resistance profiles of bacterial isolates from children 2–5 years of age with suspicion of bacterial tonsilitis at Hargeisa Group of Hospital.

Bacterial species	R1	R2	R3	R4	R5	Overall MDR
	*N* (%)	*N* (%)	*N* (%)	*N* (%)	*N* (%)	*N* (%)
Beta-hemolytic streptococci (78)	16 (20.5)	21 (26.9)	24 (30.3)	14 (17.9)	3 (3.8)	41 (52.6)
*S*. *aureus* (42)	8 (19)	16 (38)	2 (4.8)	7 (16.7)	8 (11.9)	17 (40.5)
*S*. *pneumoniae* (10)	4 (40)		5 (50)	1 (10)	0	6 (60)
*K*. *pneumoniae* (6)	1 (16.7)	2 (33.3)	1 (16.7)	2 (33.3)	0	3 (50)
*M*. *catarrhalis* (4)	1 (25)	2 (50)	1 (25)	0	0	1 (25)
*P*. *aeruginosa* (3)	0	0	2 (66.7)	1 (33.3)	0	2 (66.7)

Total (143)	30 (20.9)	41 (28.7)	35 (24.5)	25 (17.5)	8 (5.6)	71 (49.7)

*Key.* R1, R2, R3, R4, R5, and R5: non-susceptible to 1, 2, 3, 4, and 5 antibiotics categories, respectively; MDR: resistance of an isolate to three or more antibiotics taken from different categories.

**Table 7:** Multivariable analysis of factors associated with positive bacterial throat culture among children 2–5 years of age at HGH (*n* = 374).

Variables	COR (95% CI)	*P* value	AOR (95% CI)	*P* value
Gender				
Male	0.64 (0.41–1.00)	0.05	0.68 (0.38–1.19)	0.18
Female				
History of tonsillitis				
Yes	0.11 (0.68–0.19)	<0.001	01.2 (0.06–0.21)	<0.001
No				
Type of tonsillitis				
Acute				0.67
Chronic	8.37 (4.82–14.53)	<0.001		
Recurrent	0.87 (0.45–1.67)	0.67	0.45 (0.01–17.2)	
Parental occupation				
Unemployed	1.98 (1.19–3.29)	0.008	1.68 (0.89–3.18)	0.11
Employed				
Contact with cough				
Yes	1.89 (1.18–3.0)	0.008	0.71 (0.37–1.33)	0.29
No				
Tonsillar structure				
Yes	3.52 (1.93–6.42)	<0.001	2.1 (0.05–80.7)	0.69
No				
Overcrowded				
Yes	0.56 (0.35–0.89)	0.02	0.93 (0.45–1.91)	0.93
No				
Difficulty of swallowing				
Yes	0.32 (0.198–0.53)	<0.001	6.99 (3.56–13.73)	<0.001
No				
Weight loss				
Yes	2.96 (1.89–4.64)	<0.001	0.33 (0.186–0.597)	<0.001
No				
Attending school				
Yes	0.31 (0.19–0.51)	<0.001	2.98 (1.64–5.42)	<0.001
No				
Attending daycare centre				
Yes	1.54 (0.93–2.53)	0.09	1 (0.44–2.34)	0.97
No				
Wood biofuel				
Yes	7.29 (2.21–24.1)	0.001	0.19 (0.04–0.84)	0.029
No				

## Data Availability

The data used to support the findings of this study are included within the article.

## Abbreviations

AMEs: Aminoglycoside-modifying enzymes
AST: Antimicrobial sensitivity testing
ATCC: American Type Culture Collection
AOR: Adjusted odds ratio
BA: Blood agar
CA: Chocolate agar
CDC: Centre for Disease Control
CFU: Colony forming unit
CLSI: Clinical and Laboratory Standard Institute
CMHS: College of Medicine and Health Science
COR: Crude odds ratio
ENT: Ear, nose, and throat
HGH: Hargeisa Group of Hospital
IRB: Institutional Review Board
MAC: MacConkey Agar
MDR: Multidrug resistant
MHA: Mueller-Hinton agar
MRSA: Methicillin resistant *Staphylococcus aureus*
MSA: Manitol salt agar
QRDR: Quinolones resistance-determining region
SPSS: Statistical Software Package of Social Science.
